# Mentored peer review of standardized manuscripts as a teaching tool for residents: a pilot randomized controlled multi-center study

**DOI:** 10.1186/s41073-017-0032-0

**Published:** 2017-06-05

**Authors:** Victoria S. S. Wong, Roy E. Strowd, Rebeca Aragón-García, Yeseon Park Moon, Blair Ford, Sheryl R. Haut, Joseph S. Kass, Zachary N. London, MaryAnn Mays, Tracey A. Milligan, Raymond S. Price, Patrick S. Reynolds, Linda M. Selwa, David C. Spencer, Mitchell S. V. Elkind

**Affiliations:** 1 Department of Neurology, Oregon Health and Science University, Portland, OR USA; 2 Department of Neurology, Wake Forest Baptist Medical Center, 1 Medical Center Blvd, Winston-Salem, NC 27157 USA; 3 Department of Neurology, Columbia University College of Physicians and Surgeons, 630 W 168th St, New York, NY 10032 USA; 4 Department of Neurology, Montefiore Medical Center, Albert Einstein College of Medicine, 1300 Morris Park Avenue, Bronx, NY 10461 USA; 5 Department of Neurology, Baylor College of Medicine, 1 Baylor Plaza, Houston, TX 77030 USA; 6 Department of Neurology, University of Michigan, 500 S State St, Ann Arbor, MI 48109 USA; 7 Department of Neurology, Cleveland Clinic, 9500 Euclid Avenue, Cleveland, OH 44195 USA; 8 Department of Neurology, Brigham and Women’s Hospital, 75 Francis St, Boston, MA 02115 USA; 9 Department of Neurology, University of Pennsylvania, 3400 Spruce St, Philadelphia, PA 19104 USA; 10 Department of Epidemiology, Columbia University Mailman School of Public Health, 722 W 168th St, New York, NY 10032 USA; 11 The Queens Medical Center Neuroscience Institute, 1301 Punchbowl St., QET5, Honolulu, HI 96813 USA

**Keywords:** Peer review, Training, Education, Medical residency

## Abstract

**Background:**

There is increasing need for peer reviewers as the scientific literature grows. Formal education in biostatistics and research methodology during residency training is lacking. In this pilot study, we addressed these issues by evaluating a novel method of teaching residents about biostatistics and research methodology using peer review of standardized manuscripts. We hypothesized that mentored peer review would improve resident knowledge and perception of these concepts more than non-mentored peer review, while improving review quality.

**Methods:**

A partially blinded, randomized, controlled multi-center study was performed. Seventy-eight neurology residents from nine US neurology programs were randomized to receive mentoring from a local faculty member or not. Within a year, residents reviewed a baseline manuscript and four subsequent manuscripts, all with introduced errors designed to teach fundamental review concepts. In the mentored group, mentors discussed completed reviews with residents. Primary outcome measure was change in knowledge score between pre- and post-tests, measuring epidemiology and biostatistics knowledge. Secondary outcome measures included level of confidence in the use and interpretation of statistical concepts before and after intervention, and RQI score for baseline and final manuscripts.

**Results:**

Sixty-four residents (82%) completed initial review with gradual decline in completion on subsequent reviews. Change in primary outcome, the difference between pre- and post-test knowledge scores, did not differ between mentored (−8.5%) and non-mentored (−13.9%) residents (*p* = 0.48). Significant differences in secondary outcomes (using 5-point Likert scale, 5 = strongly agree) included mentored residents reporting enhanced understanding of research methodology (3.69 vs 2.61; *p* = 0.001), understanding of manuscripts (3.73 vs 2.87; *p* = 0.006), and application of study results to clinical practice (3.65 vs 2.78; *p* = 0.005) compared to non-mentored residents. There was no difference between groups in level of interest in peer review (3.00 vs 3.09; *p* = 0.72) or the quality of manuscript review assessed by the Review Quality Instrument (RQI) (3.25 vs 3.06; *p* = 0.50).

**Conclusions:**

We used mentored peer review of standardized manuscripts to teach biostatistics and research methodology and introduce the peer review process to residents. Though knowledge level did not change, mentored residents had enhanced perception in their abilities to understand research methodology and manuscripts and apply study results to clinical practice.

**Electronic supplementary material:**

The online version of this article (doi:10.1186/s41073-017-0032-0) contains supplementary material, which is available to authorized users.

## Background

With an ever-expanding body of scientific literature [1] and peer reviewers in demand [2], there is a need to train potential peer reviewers to keep up with the growing need. Simultaneously, there is a continued emphasis from the Accreditation Council for Graduate Medical Education (ACGME) to train residents to appraise and assimilate scientific evidence from the biomedical literature [3]. Prior studies have found that formal education in biostatistics and research methodology during residency is lacking [4–7]. By training residents in the peer review process, there is potential to develop a larger pool of peer reviewers while providing them with the skills necessary to interpret and produce scholarly works that impact patient care.

In this current pilot study, we evaluated a novel method of teaching neurology residents the basic concepts of biostatistics, research methodology, and review of scholarly literature employing a program of peer review of scientific manuscripts. We hypothesized that mentored peer review of standardized manuscripts is feasible and would improve resident perception and knowledge of the principles of biostatistics and research methodology more than non-mentored peer review.

## Methods

### Study sites and population

A partially blinded, randomized, controlled multi-center pilot study of mentored peer review of standardized manuscripts was performed. Through the American Academy of Neurology (AAN) Consortium of Neurology Program Directors, we sent an e-mail invitation to Program Directors of ACGME-accredited adult neurology residency programs in the USA to participate. Participation interest was high with 38 sites expressing preliminary interest. Nine sites were chosen based on the size of residency program (to maximize the number of resident participants) and the sites at which study investigators were affiliated. Study sites included the Montefiore Medical Center, Albert Einstein College of Medicine; Baylor College of Medicine; Brigham and Women’s Hospital and Massachusetts General Hospital (Partners); Cleveland Clinic; Columbia University; Oregon Health & Science University; University of Michigan; University of Pennsylvania; and Wake Forest Baptist Medical Center. We recruited adult neurology residents in their post-graduate years (PGY) 3 and 4 from the nine chosen study sites. Volunteer mentors were recruited locally from existing faculty at each site. An outline of the curriculum and brief guidelines were provided to each faculty mentor. The curriculum outline is available in an additional file (see Additional file 1).

### Human subject protection

The institutional review boards at each participating study site either approved or exempted the research protocol as an education research study, based on local regulations and criteria. All resident participants provided written informed consent as required by their study site.

### Study design

Program directors received an introductory packet including study overview and the brief curriculum mentioned above. Program directors and coordinators were instrumental in study coordination and facilitating pre- and post-test administration. All consenting neurology resident participants took an initial pre-test assessing knowledge of biostatistics and research methodology (see “Survey instruments and knowledge tests” below). Prior to receiving any manuscripts for review, all residents received a document with basic tips on effective peer review. They were also each given the reference textbook *Clinical Epidemiology: The Essentials* [8].

### Blinding and allocation concealment

Residents were randomized (1:1) to mentored and non-mentored groups within each site by a research assistant who used a computerized random number generator to perform the group allocation.

### Intervention

**Fig. 1:**
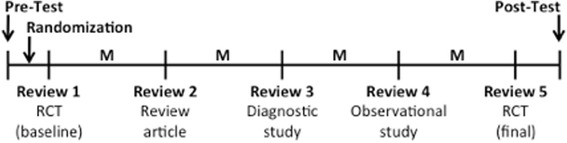
Study schema. Abbreviations: *M* mentor meeting (for those randomized to the mentored group), *RCT* randomized controlled trial

Each mentored resident had one mentor. Residents were given a baseline standardized manuscript (manuscript 1) for peer review (see “Standardized manuscripts” below) to serve as a baseline (Fig. 1). Their randomization status (mentored vs non-mentored) was revealed to them after completion of the first review. Mentors were advised to discuss the reviews with residents after completion of each manuscript review, focusing on key teaching points outlined in the program curriculum for each manuscript. For the mentored group, meetings with the mentor were mandatory after completion of each of the first four manuscripts, though compliance was not enforced. Residents in the non-mentored group were not assigned a faculty mentor but continued to receive manuscripts and were expected to complete reviews.

Including the baseline manuscript (manuscript 1), residents were given a total of five manuscripts for review at two-month intervals, with the study period lasting from 2012 to 2013. Each resident participant as well as the faculty mentors received the same sets of standardized manuscripts. Residents had a 6-week period to complete the manuscript review and meet with their mentor, if applicable. There was a 2-week grace period per review period, and late reviews were accepted.

### Standardized manuscripts

The five standardized manuscripts incorporated deliberate errors, using a previously published methodology [9]. Manuscripts from 2004 to 2007 were chosen, with permission from the *Neurology®* journal, to minimize the chance that residents would have read these articles while in training [10–14]. Manuscripts were chosen to represent different experimental designs, including randomized controlled trials (manuscripts 1 and 5, allowing for comparison of review quality) [10, 11], a review (manuscript 2) [12], a diagnostic accuracy study (manuscript 3) [13], and an observational study (manuscript 4) [14].

Ten deliberate errors per manuscript were introduced to adhere to a standardized curriculum of topics related to biostatistics, and research methodology after permission was obtained from the corresponding authors. The entire list of introduced manuscript errors is available as a separate file (see Additional file 2). Deliberate subversion of reporting guidelines including CONSORT [15], PRISMA [16], STARD [17], and STROBE [18] was performed to create these deliberate errors.

### Survey instruments and knowledge tests

Demographic and educational history data, including experiences likely to influence review quality and knowledge, were collected at enrollment (e.g., age, sex, primary academic degree[s], other degrees, research experience, peer reviewing experience, location of residency, prior training in research methods, and prior training in evidence-based medicine). Pre-test and post-test questions reflecting knowledge of epidemiology and biostatistics were obtained from two published studies [5, 6]. Each test contained 20 questions, without repetition of questions.

Residents’ perception of their understanding of biostatistics, level of confidence about use and interpretation of statistical concepts, and application of scientific study results to patient care were also assessed on a 5-point Likert scale (1 = strongly disagree to 5 = strongly agree) at baseline and study end. Residents were also asked to provide reasons for difficulty completing reviews and to evaluate their experience in the study.

### Evaluation of peer review quality

The quality of manuscript reviews was measured with the Review Quality Instrument (RQI), a validated instrument used to measure the quality of peer reviews in prior studies [19]. The RQI assesses whether reviews cover major important points in a research article using a 5-point Likert scale, including importance of the research question, originality, strengths and weaknesses, and interpretation. It also assesses whether the reviewer made comments on organization and writing, referenced examples within the paper, and provided constructive criticism. The first seven questions of the RQI encompass each of these points, while the eighth question assesses the overall review quality.

The RQI was used by two of the study authors (VSSW, MSVE) to assess review quality of manuscript 1 (baseline measure) and manuscript 5 (post-intervention assessment); study authors were blinded to group and identity.

### Outcome measures

The primary outcome measure was the change in knowledge between the pre-test and the post-test performed after review of all manuscripts. Secondary outcome measures included level of confidence in the use and interpretation of statistical concepts before and after the intervention, as well as the RQI score for the baseline (manuscript 1) and final (manuscript 5) manuscripts. Both manuscript 1 and manuscript 5 were randomized controlled trials, allowing for comparison of review quality.

### Statistical analysis

Demographics and educational experiences at enrollment were compared using chi-square tests for categorical measures and Wilcoxon rank-sum tests for continuous measures. Results were summarized as means (± standard deviation) and proportions (%) as appropriate. Primary and secondary outcome measure scores were compared using ANCOVA or Wilcoxon rank-sum tests as appropriate for the two groups (with and without mentorship). For the primary outcome of the change in knowledge based on pre- and post-test scores, the change was calculated only among those with both tests completed and compared accounting for pre-test scores. We also tested whether pre-test score was dependent on missing the post-test using logistic regression with indication of missingness (1 for post-test not done and 0 for post-test done) as a dependent variable and pre-test as an independent variable. For other outcomes, we analyzed all available data. The average score of two reviewers was used for the RQI outcome. The inter-rater reliability of the RQI between the two reviewers was tested using the intra-rater correlation coefficient (ICC). Software used for analysis was SAS version 9.3 (SAS Institute, Cary, NC).

## Results

**Fig. 2:**
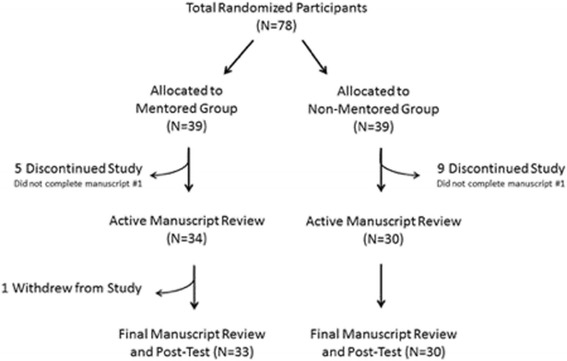
Flow diagram

A total of 78 residents from 9 neurology programs were enrolled and randomized to the mentored (*n* = 39, 50%) and non-mentored (*n* = 39, 50%) groups. Fourteen residents withdrew from the study, 5 mentored and 9 non-mentored (Fig. 2). Baseline characteristics were similar between groups, though time from medical school was slightly longer in the non-mentored group (Table 1). Both mentored and non-mentored residents were likely to report previously having a mentor (79 and 95%, respectively), with a trend toward more baseline mentoring in the non-mentored group (Table 1). Approximately half of the participants reported receiving prior teaching in epidemiology (*n* = 35, 45%), biostatistics (*n* = 38, 49%), or evidence-based medicine (*n* = 48, 62%). The majority of residents reported reading scientific journals (*n* = 76, 97%) and participating in prior research (*n* = 67, 86%).

**Table 1:** Participant characteristics

Demographics (*n*)	Total (78)	Non-mentored (39)	Mentored (39)
Age, years, mean (STD)	30.8 (2.6)	30.2 (2.2)	31.3 (2.8)
Male sex, *n* (%)	39 (50%)	21 (54%)	18 (46%)
Advanced degrees, *n* (%)			
- MD	57 (73%)	28 (72%)	29 (74%)
- DO	2 (3%)	1 (3%)	1 (3%)
- Additional Graduate degree	18 (23%)	10 (25.6%)	9 (23%)
Years since medical school, mean (STD)	3.4 (1.7)	3.7 (2.0)	3.1 (1.3)
Year in training, *n* (%)			
- PGY3	37 (47%)	22 (56%)	15 (38%)
- PGY4^a^	41 (53%)	17 (44%)	24 (62%)
Prior epidemiology education, *n* (%)	35 (45%)	16 (41%)	19 (49%)
Prior biostatistics education, *n* (%)	38 (49%)	21 (54%)	17 (44%)
Prior evidence-based medicine education, *n* (%)	48 (62%)	27 (69%)	21 (54%)
Reads scientific journals, *n* (%)	76 (97%)	38 (97%)	38 (97%)
Participated in research, *n* (%)	67 (86%)	33 (85%)	34 (87%)
No. prior publications, *n* (%)			
- 0	32 (41%)	16 (41%)	16 (41%)
- 1–5	39 (50%)	19 (49%)	20 (51%)
- >5	7 (9%)	4 (10%)	3 (8%)
Current faculty mentor, *n* (%)	68 (87%)	37 (95%)	31 (79%)
Baseline perceptions of biostatistics: 5-point Likert score (1 = strongly disagree to 5 = strongly agree)			
“Would like to learn more about biostatistics,” mean (STD)	4.38 (0.84)	4.44 (0.85)	4.33 (0.84)
“Can understand almost all of statistical terms in journal articles,” mean (STD)	2.68 (0.93)	2.64 (0.99)	2.72 (0.89)
“I do not trust statistics,” mean (STD)	2.4 (0.87)	2.54 (0.82)	2.34 (0.91)
“I use statistical information in medical care,” mean (STD)	3.54 (0.94)	3.49 (0.91)	3.59 (0.97)
“Necessary to know about statistics,” mean (STD)	4.71 (0.54)	4.64 (0.63)	4.77 (0.43)
Confidence in ability to, mean (STD)			
- Interpret *p* values	3.73 (0.82)	3.82 (0.76)	3.64 (0.87)
- Interpret statistical methods	2.71 (0.69)	2.74 (0.75)	2.67 (0.62)
- Assess if correct statistical procedure used	1.96 (0.81)	1.95 (0.76)	1.97 (0.87)
- Identify factors influencing study power	2.45 (0.77)	2.41 (0.88)	2.49 (0.64)
- Apply study results to clinical practice	2.96 (0.69)	2.97 (0.67)	2.95 (0.7)

*Abbreviation: STD* standard deviation

^a^Includes 1 PGY-5 in pediatric neurology

Baseline perceptions of level of knowledge and confidence in biostatistics were well matched between groups (Table 1). Residents strongly agreed that they would like to learn more about biostatistics and that it was necessary to know about statistics to intelligently interpret the medical literature. Confidence in understanding statistical terms, interpreting *p* values, interpreting statistical methods, assessing whether the correct statistical procedure was used in a study, identifying factors influencing study power, and applying study results to clinical practice were all low and not statistically different between groups (all *p* > 0.40).

Participation was high initially with 64 (82%) residents completing review of manuscript 1; there was a gradual decline over time (49 [63%] completed review of manuscript 2, 35 [45%] completed manuscript 3, 29 [42%] completed manuscript 4, and 44 [56%] completed manuscript 5; Table 2 (A)). The investigators strongly encouraged residents to complete manuscript 5. The majority of residents (*n* = 46, 59%) completed at least 3 of 5 manuscript reviews. There was no difference between groups in the overall number of reviews completed (Table 2 (B)). The most frequently reported impediment to manuscript review was the residents’ busy schedules, with 65% (*n* = 30) reporting this as the primary reason for not completing a manuscript review.

**Table 2:** Reviews completed by all enrolled participants

Part A: specific manuscript reviews completed	Total, *n* = 78	Non-mentored, *n* = 39	Mentored, *n* = 39	
Manuscript 1: randomized controlled trial	64 (82%)	30 (47%)	34 (53%)	
Manuscript 2: systematic review	49 (63%)	26 (53%)	23 (47%)	
Manuscript 3: diagnostic accuracy study	35 (45%)	18 (51%)	17 (49%)	
Manuscript 4^a^: observational study	29 (42%)	14 (48%)	15 (52%)	
Manuscript 5: randomized controlled trial	44 (56%)	24 (55%)	20 (45%)	
Part B: total number of reviews completed	Total (%)	Non-mentored	Mentored	*p* value^†^
No reviews completed	14 (18.0)	9 (64%)	5 (36%)	0.238
1 review	10 (12.8)	2 (20%)	8 (80%)	0.0421
2 reviews	8 (10.3)	4 (50%)	4 (50%)	1
3 reviews	10 (12.8)	5 (50%)	5 (50%)	1
4 reviews	15 (19.2)	8 (53%)	7 (47%)	0.77
5 reviews	21 (26.9)	11 (52%)	10 (48%)	0.799

^a^9 students (5 non-mentored, 4 mentored) only received 4 manuscripts and did not complete manuscript 4

^†^Chi-squared with 1 degrees of freedom test

Twenty-five of the 39 residents (64%) who were randomized to the mentored group responded to the post-intervention questions on the frequency of mentor meetings. The 25 who responded reported a mean of 2.8 ± 1.2 out of 4 expected mentor-mentee meetings and a median of 3 meetings. Five (20%) completed one meeting, 5 (20%) completed two meetings, 6 (24%) completed three meetings, 8 (32%) completed four meetings, and 1 (4%) completed five meetings (though we did not request a meeting after the final manuscript review). Fifteen (60%) completed three or more mentor meetings.

Several barriers to meeting with mentors were expressed, but the most often cited (*n* = 12, 71%) was the busy resident schedule. An additional table reporting barriers to review completion and mentor meetings, as well as desire for mentorship is available in a separate file (see Additional file 3). Overall, residents indicated a strong interest in being mentored, with 82% (*n* = 40) indicating that they would desire mentoring in future similar studies. Mentored residents were more likely to feel indifferent about the benefits of mentoring compared to non-mentored residents (24 vs 8%).

### Primary outcome measure

Pre-test knowledge scores were 66.0 ± 14.7% correct, and post-test scores were 54.9 ± 12.1% correct (Table 3 (A)). Seventy-eight residents took the initial pre-test, of whom 51 completed the post-test. Pre-test scores did not differ between those with both test done vs. only pre-test done (*p* = 0.85). Scores trended similarly in mentored and non-mentored residents and were not different at follow-up (*p* = 0.14). The mentored group had less decline in the change between pre-test and post-test compared to the non-mentored group, though no significant difference was found (*p* = 0.48).

**Table 3:** Knowledge, Review Quality Instrument, and study perception

	Total	Non-mentored	Mentored	*p* value^*^
Part A: knowledge questions				
Pre-test, mean % correct (STD), *n*	66.0 (14.7), *n* = 51	67.8 (14.2), *n* = 25	64.2 (15.3), *n* = 26	0.65
Post-test, mean % correct (STD), *n*	54.9 (12.1), *n* = 51	53.9 (11.8), *n* = 25	55.8 (12.3), *n* = 26	0.14^**^
Change in score, % decline (STD), *n*	−11.1 (17.5), *n* = 51	−13.9 (16.7), *n* = 25	−8.5 (18.2), *n* = 26	0.48^**^
Part B: overall study perception, 5-point Likert score from 1 = strongly disagree to 5 = strongly agree (STD)				
Participation has enhanced:				
- Understanding of research methodology	3.18 (1.1)	2.61 (1.2)	3.69 (0.8)	0.001
- Interest in clinical research	3.08 (1.1)	2.83 (1.2)	3.31 (1.0)	0.108
- Understanding of manuscripts	3.33 (1.1)	2.87 (1.3)	3.73 (0.9)	0.006
- Motivation to read more manuscripts	3.12 (1.1)	2.78 (1.2)	3.42 (0.9)	0.075
- Interest in peer review	3.04 (1.0)	3.09 (0.9)	3.00 (1.1)	0.72
- Interest in academic neurology	3.00 (1.0)	2.83 (1.0)	3.15 (1.1)	0.375
- Application of study results to clinical practice	3.25 (1.1)	2.78 (1.1)	3.65 (0.9)	0.005
- Explanation of studies to patients	2.90 (1.2)	2.43 (1.3)	3.31 (0.9)	0.010
- Overall impression	3.39 (1.1)	3.22 (1.1)	3.54 (1.0)	0.279
- Enjoyment in participating in research study	3.18 (1.1)	3.17 (1.1)	3.19 (1.1)	0.925
Part C: RQI scores				
Manuscript 1, mean (STD)				
- Mean of Q1–7	3.46 (0.8)	3.38 (0.9)	3.53 (0.7)	0.56
- Mean of Q8	3.48 (0.9)	3.45 (1.1)	3.51 (0.8)	0.92
Manuscript 5, mean (STD)				
- Mean of Q1–7	3.15 (0.84)	3.06 (0.97)	3.25 (0.7)	0.50
- Mean of Q8	3.31 (1.0)	3.13 (1.1)	3.53 (0.8)	0.19

*Abbreviations: STD* standard deviation, *RQI* Review Quality Index, *Q* question

^*^Wilcoxon rank-sum test

^**^ANCOVA adjusting for pre-test scores

### Secondary outcome measures

After intervention, mentored residents perceived enhanced experiences compared to non-mentored residents in several topics, including understanding research methodology (*p* = 0.001), understanding of manuscripts (*p* = 0.006), application of study results to clinical practice (*p* = 0.005), and explanation of studies to patients (*p* = 0.010). No difference was observed between groups in the overall perception of the quality of their experience in the program (Table 3 (B)).

The inter-rater reliability of the RQI was good (ICC = 0.7 for the two independent reviewers). The quality of manuscript review as assessed by the RQI did not differ between groups (Table 3 (C)).

## Discussion

This pilot study demonstrates the use of an innovative approach to teaching peer review. Mentoring did not impact either the quality of peer review or knowledge of biostatistics, though it impacted perceived knowledge and confidence in understanding of research methodology, scientific manuscripts, and application of scientific data to clinical practice and patient care.

Peer review quality was assessed in this study using the RQI. No difference in review quality was found between the baseline and final manuscript review. Numerous prior attempts at training peer reviewers, including the use of training workshops [9, 20, 21], self-taught training packages [9], and written feedback from journal editors [22], have not yielded sustained improvements in peer review quality. To our knowledge, this is the first study using the RQI to evaluate review quality in a trainee population (i.e., residents). Further studies will be needed to test whether more structured approaches to mentoring, training of mentors, or supplementing with a formal curriculum on biostatistics for residents can improve peer review quality.

Residency training is an important time to teach, develop, and reinforce skills in critical appraisal of medical literature and implementation of scientific data to patient care. Numerous curricula have been explored to teach the important principles underlying biostatistics, research methodology, and evidence-based medicine [23, 24]. Our novel approach integrates clinically relevant studies which impact neurologic patient care with an exposure to peer review of manuscripts. Curricula of this type which include formal training in peer review are lacking [25].

The impact of mentoring in this study was uncertain. The role of mentorship in promoting career advancement, increasing scholarly activities, encouraging professionalism, enhancing personal growth, and improving career satisfaction is well recognized [26–28]. However, in our current study, mentoring did not appear to impact knowledge acquisition or performance. Potential explanations include the high prevalence of faculty mentoring and reading of scientific articles at baseline, as well as the trend toward higher baseline mentoring in the non-mentored group. While a general curriculum outlining goals and pertinent topics as well as an introductory epidemiology and biostatistics text was provided to each program involved in the study, formal training for mentors and a structured resident curriculum was not incorporated into our pilot study and could be considered in future studies, particularly given evidence from other studies on the positive impact of mentor training on mentoring quality [29].

Our data provide evidence that there may be positive and negative aspects to mentorship. Qualitative comments provided by participants suggested that residents in the non-mentored group expressed disappointment about not being assigned a mentor. However, residents in the mentored group were no more likely to have completed manuscript reviews compared to the non-mentored group. For those residents who were assigned a mentor, obstacles to meeting with the mentor did exist and difficulties in finding time to meet may have led residents in the mentored group to forgo review completion altogether if they felt that they would not be able to arrange a meeting.

Our study has limitations. Formal training for volunteer faculty mentors was not included and, while a curriculum of suggested topics was provided, instruction provided to each resident was not standardized. There is also an inevitable variability in mentorship quality and style, as well as variation in number of mentors across sites. Participating residents had high baseline rates of reading of scientific journals and mentorship, possibly reducing the effect size of the intervention. Mentor-mentee meetings were mandated, but compliance was not enforced, potentially diluting an effect of the study intervention. The time given to complete each manuscript may have been insufficient, resulting in reduced completion rates mid-way through the study. This mirrors real world peer review limitations, with time constraints sited as the primary reason for refusing to complete peer reviews [30]. Lastly, the sample size was small in this pilot study focusing only on neurology residents, and future consideration should be made to a wider scale study including trainees in other specialties, allowing for a greater degree of generalization.

Our study also had strengths. We incorporated a novel hands-on teaching tool in a multi-center study to address a known knowledge gap in residency training. The use of standardized manuscripts representing different study designs with systematically introduced errors provided a broad scope of teaching points, so that the residents could learn more about different aspects of biostatistics and research methodology. Additional long-term follow-up data on the impact of this novel educational design on future resident scholarship are ongoing. Future directions to improve the benefit of using peer review as a teaching tool include increased guidance of faculty mentors, use of more specialized but remote mentors [31] (e.g., established editors of scientific journals with an interest in peer review), focus on a more formal curriculum, web-based tutorials, fewer manuscript assignments, and the potential use of live submitted manuscripts rather than standardized ones to provide a more realistic review experience. These changes may pique resident interest and encourage continued participation.

## Conclusions

In conclusion, we used mentored peer review of standardized manuscripts to introduce the concept of peer review and to teach the principles of biostatistics and research methodology to neurology residents. Although primary outcome measure of content knowledge did not increase, mentored residents had an enhanced perception in their abilities to understand research methodology and scientific manuscripts as well as their ability to apply study results to clinical practice and explain these to patients. Future studies with a larger sample size, incorporating formal mentoring training and a more detailed curriculum, may enhance the impact of this education intervention and its application to both the peer review process and patient care.

## Additional files

Additional file 1: Program curriculum outline. (DOCX 74 kb)

Additional file 2: Introduced manuscript errors. (DOCX 113 kb)

Additional file 3: Mentorship impressions. (DOCX 70 kb)
