# Supplementary material for: Mentored peer review of standardized manuscripts as a teaching tool for residents: a pilot randomized controlled multi-center study
Source: Res Integr Peer Rev. 2017 Jun 5;2:6. doi: 10.1186/s41073-017-0032-0 (PMC5803578; doi:10.1186/s41073-017-0032-0)
Supplement: Supplementary file 1 — Program curriculum outline. (DOCX 74 kb) [file 41073_2017_32_MOESM1_ESM.docx]

**Supporting Information 1: Program Curriculum**

The following is an overview of topics with the potential to be reviewed during this study. We encourage that this curriculum be distributed to mentors and that these topics be considered as areas of potential discussion during the face-to-face mentoring sessions.

General Biostatistics

- P-values
- Sample size and power
- Chance and type I/II errors
- Chi-squared test
- Student’s t-test
- ANOVA
- Correlation and regression

Prevention and Therapy

- Randomized controlled trials
- Blinding
- Intention to treat
- Relative risk, absolute risk, relative/absolute risk reduction
- Number needed to treat
- Co-interventions

Prognosis

- Cohort study, case control study, case series
- Confounding and bias
- Risk ratios, odds ratios
- Kaplan-Meier analysis

Diagnosis / Screening Test

- Diagnostic accuracy study
- Reference / gold standard
- Incidence, prevalence
- Sensitivity, specificity
- Likelihood ratios
- Pre- and post-test probability

Systematic review

- Reviews / meta-analyses
- Standard deviation
- Confidence intervals
- Heterogeneity, homogeneity
