# Supplementary material for: Mentored peer review of standardized manuscripts as a teaching tool for residents: a pilot randomized controlled multi-center study
Source: Res Integr Peer Rev. 2017 Jun 5;2:6. doi: 10.1186/s41073-017-0032-0 (PMC5803578; doi:10.1186/s41073-017-0032-0)
Supplement: Supplementary file 2 — Introduced manuscript errors. (DOCX 113 kb) [file 41073_2017_32_MOESM2_ESM.docx]

**Supporting Information 2: Introduced Manuscript Errors**

***Manuscript 1:*** Treatment of pseudobulbar affect in ALS with dextromethorphan/quinidine: a randomized trial^10^

***Study design:*** randomized controlled trial

***Reporting guideline:*** CONSORT^15^

***Errors introduced:***

1. Invalid rationale for the study, CONSORT item 2
   - Deleted study justification.
2. Lack of generalizability, CONSORT items 2, 21
   - Changed from multi-center to single-center trial.
3. No IRB approval statement, CONSORT item 4
   - Removed IRB approval statement.
4. No validation of rating scales, CONSORT item 12
   - Removed any mention of validation of rating scales used.
5. Lack of exclusion or eligibility criteria, CONSORT items 3, 4
   - Removed exclusion and eligibility criteria.
6. Inappropriate randomization, CONSORT item 8
   - Reported that study participants were grouped by name alphabetization instead of randomized.
7. Unblinded study, CONSORT item 11
   - Added a statement suggesting that the study was not blinded.
8. Inadequate adverse event monitoring, CONSORT items 3, 19
   - Reported that only a proportion of the study participants were monitored for adverse effects.
9. Inadequate statistical analysis, CONSORT items 13, 20
   - Failed to explain why an outlier was excluded.
10. Unjustified conclusion, CONSORT items 21, 22
    - Concluded widespread generalizability from a single center trial.

***Manuscript 2:*** Ethnic variation in the incidence of ALS: a systematic review^12^

***Study design:*** systematic review

***Reporting guideline:*** PRISMA^16^

***Errors introduced:***

1. Non-standard search strategy, PRISMA item 8
   - Reported that an inadequate Google Scholar search was performed without standardized search terms.
2. Biased data extraction, PRISMA item 10
   - Added a biased data extractor (business manager at an ALS center).
3. Biased study selection, PRISMA items 9, 12
   - Included all studies from a single study population.
4. Selective reporting, PRISMA item 7
   - Did not include additional references from article citations in the study.
5. Appropriate regression analyses not performed, PRISMA items 15, 16
   - Removed adjustment for age.
6. Limitations not discussed, PRISMA item 25
   - Removed discussion of limitations.
7. Within-study bias, PRISMA item 19
   - Misinterpreted one of the reported studies with subsequent discussion removed.
8. Confidence intervals not reported, PRISMA item 21
   - Removed all confidence intervals in the manuscript.
9. Incorrect interpretation of results, PRISMA items 24, 26
   - Over-interpreted results, removing nuance.
10. Inappropriate determination of heterogeneity, PRISMA items 14, 24
    - Focused case ascertainment at large medical centers only, to improve study homogeneity.

***Manuscript 3:*** What does fluorodeoxyglucose PET imaging add to a clinical diagnosis of dementia?^13^

***Study design:*** diagnostic accuracy study

***Reporting guideline:*** STARD^17^

***Errors introduced:***

1. Invalid interpretation of existing literature, STARD item 2
   - Interpreted existing literature incorrectly.
2. Assessment dates not provided, STARD items 6, 14
   - Removed assessment dates from study.
3. Assessment bias, STARD item 10
   - Reported use of an inappropriate examiner (research assistant) to interpret studies.
4. Inadequate definition of condition, STARD item 3
   - Reported inadequate study participant selection based on ICD-9 coding.
5. Inadequate exclusion criteria, STARD item 16
   - Reported that exclusion of study participants was based on clinician judgment.
6. Non-standard assessment, STARD item 7
   - Reported assessment as use of radiology reports rather than direct review of images.
7. Lack of reproducibility, STARD items 24, 25
   - Reported that autopsy protocols were variable.
8. Invalid explanation of patient exclusion, STARD items 16, 20
   - Reported that study participants were excluded for not completing tests or having tests with uninterpretable results, without further explanation given.
9. Standard deviations not reported, STARD item 21
   - Removed standard deviation data.
10. Invalid conclusion, STARD item 25
    - Suggested inappropriately that the study is the new gold standard for diagnosis.

***Manuscript 4:*** Rate, degree, and predictors of recovery from disability following ischemic stroke^14^

***Study design:*** observational study

***Reporting guideline:*** STROBE^18^

***Errors introduced:***

1. Undefined study objectives, STROBE item 3
   - Deleted study objectives.
2. Unspecified study design and setting, STROBE items 4, 5
   - Removed background information about study cohort.
3. Inappropriate study design, STROBE items 11, 12
   - Used inappropriate quantitative variables / time intervals for statistical method.
4. Invalid data exclusion, STROBE item 12
   - Excluded interview data inappropriately without providing comparable alternative for study participants who could not complete the interview due to medical condition.
5. Confidence intervals not reported, STROBE item 16
   - Removed confidence intervals.
6. Inadequate follow-up, STROBE item 13
   - Reported follow-up time points at only 1 month and 18 months.
7. Lack of adjustment for confounders, STROBE item 16
   - Removed some covariates.
8. Incomplete analysis, STROBE item 17
   - Extrapolated some study outcome data rather than measuring directly.
9. Study limitations not discussed, STROBE item 19
   - Removed study limitations.
10. Lack of generalizability, STROBE item 21
    - Reported over-reaching conclusion implying generalizability.

***Manuscript 5:*** Petasites hybridus root (butterbur) is an effective preventive treatment for migraine^11^

***Study design:*** randomized controlled trial

***Reporting guideline:*** CONSORT^15^

***Errors introduced:***

1. Invalid trial design, CONSORT item 3
   - Removed specification of whether study was a single- or multi-center study.
2. Lack of eligibility criteria, CONSORT item 4
   - Deleted eligibility criteria.
3. Inability to replicate study, CONSORT item 5
   - Deleted detail about the medication’s active ingredient that would be necessary to replicate the study.
4. Unspecified outcome measures, CONSORT item 6
   - Didn’t specify primary or secondary outcome measures.
5. Lack of power calculation, CONSORT item 7
   - Deleted power calculation.
6. No intention-to-treat analysis, CONSORT item 16
   - Removed intention-to-treat analysis.
7. Unspecified reasons for withdrawal from study, CONSORT item 13
   - Removed reasons for participant withdrawal from study.
8. No baseline data table, CONSORT item 15
   - Removed baseline characteristics (Table 1).
9. Adverse effects in placebo group not measured, CONSORT item 19
   - Removed report of adverse effects in placebo group.
10. Invalid study interpretation, CONSORT item 22
    - Made inappropriate cross-study conclusions.
