# Supplementary material for: Mentored peer review of standardized manuscripts as a teaching tool for residents: a pilot randomized controlled multi-center study
Source: Res Integr Peer Rev. 2017 Jun 5;2:6. doi: 10.1186/s41073-017-0032-0 (PMC5803578; doi:10.1186/s41073-017-0032-0)
Supplement: Supplementary file 3 — Mentorship impressions. (DOCX 70 kb) [file 41073_2017_32_MOESM3_ESM.docx]

**Supporting Information 3**

***Table: Mentorship Impressions***

|  | Total | Non-Mentored | Mentored |
| --- | --- | --- | --- |
| What impeded manuscript review the most   - No impediments - Busy residency schedule - Insufficient time provided - Lack of interest - Other | 14 (30%)  30 (65%)  1 (2%)  5 (11%)  1 (2%) | 6 (27%)  16 (73%)  0 (0%)  3 (14%)  1 (5%) | 8 (33%)  14 (58%)  1 (4%)  2 (8%)  0 (0%) |
| No. of mentor-mentee meetings (mean, STD) | NA | NA | 2.8 (1.2) |
| Reasons for not meeting with mentor  (all that apply)   - Busy residency schedule - Busy mentor schedule - Insufficient time provided - Mentor meeting not helpful - Other | NA | NA | 12 (71%)  1 (6%)  2 (12%)  3 (18%)  1 (6%) |
| Would recommend future mentoring   - Want mentor - Don’t want mentor - Indifferent | 40 (82%)  1 (2%)  8 (16%) | 22 (92%)  0 (0%)  2 (8%) | 18 (72%)  1 (4%)  6 (24%) |

Abbreviations: STD = standard deviation
